# Seroincidence Rate of Typhoidal *Salmonella* in Children, Kenya, 2017–2018 

**DOI:** 10.3201/eid3203.250469

**Published:** 2026-03

**Authors:** Aslam Khan, Polina Kamenskaya, Izabela Rezende, Francis M. Mutuku, Bryson Ndenga, Zainab Jembe, Priscilla Maina, Philip Chebii, Charles Ronga, Victoria Okuta, Denise O. Garrett, Donal Bisanzio, Kristen Aiemjoy, Jason R. Andrews, A. Desiree LaBeaud, Richelle Charles

**Affiliations:** Stanford University School of Medicine, Stanford, California, USA (A. Khan, I. Rezende, J.R. Andrews, D. LaBeaud); Massachusetts General Hospital, Boston, Massachusetts, USA (P. Kamenskaya, R. Charles); Technical University of Mombasa, Mombasa, Kenya (F.M. Mutuku); Kenya Medical Research Institute, Kisumu, Kenya (B. Ndenga, C. Ronga, V. Okuta); Msambweni County Referral Hospital, Msambweni, Kenya (Z. Jembe, P. Maina, P. Chebii); Albert B. Sabin Vaccine Institute, Washington, DC, USA (D.O. Garrett); RTI International, Research Triangle Park, North Carolina, USA (D. Bisanzio); University of California, Davis, California, USA (K. Aiemjoy); Mahidol University, Bangkok, Thailand (K. Aiemjoy); Harvard Medical School, Boston (R. Charles); Harvard T.H. Chan School of Public Health, Boston (R. Charles)

**Keywords:** typhoid, bacteria, enteric infections, seroincidence, Salmonella, Kenya, children, pediatrics, infection, serocalculator, vector-borne infections

## Abstract

Enteric fever, caused by *Salmonella enterica* serovars Typhi and Paratyphi, results in high rates of illness and death globally. The lack of reliable diagnostic assays limits surveillance, leading to major gaps in understanding the population-level burden in low- and middle-income countries. We applied a novel serologic tool measuring IgG responses to hemolysin E to assess typhoidal *Salmonella* infection rates in children from 4 communities: 2 in western Kenya (Kisumu and Chulaimbo) and 2 in coastal Kenya (Ukunda and Msambweni). We found a substantially higher enteric fever seroincidence rate in coastal Kenya (37/100 person-years) than in western Kenya (3.6/100 person-years). We found a higher seroincidence rate in households with nonpiped water and lower incomes and in neighborhoods with higher population density. Our findings contribute to Kenya's limited enteric fever surveillance data, especially in the coastal regions. Such information underscores the need for public health interventions, such as typhoid conjugate vaccine introduction, in Kenya.

Enteric fever is a major health problem globally that has the potential to cause a spectrum of symptoms, including severe febrile illness and intestinal perforation (*1*). *Salmonella enterica* serovars Typhi and Paratyphi A, B, and C are responsible for enteric fever; *Salmonella* Typhi is the most prevalent, followed by *Salmonella* Paratyphi A. Whereas *Salmonella* Typhi is found throughout the world, *Salmonella* Paratyphi A is most prevalent in South and Southeast Asia and is not commonly found in Africa. Most illness occurs in low and middle-income countries (LMICs) that lack access to safe drinking water and improved sanitation; children and adolescents bear the highest burden of disease (*2*–*4*). Accurate diagnosis is challenging and often requires culture-based methods (*5*). The current reference standard is blood culture, which might be unavailable in many clinical settings where typhoid is endemic; it has an estimated sensitivity range of 51%–65% that varies by age, duration of symptoms, use of antimicrobial drugs before testing, and volume of blood collected (*5*,*6*). Alternative molecular testing using peripheral blood has low diagnostic sensitivity (*7*). Point-of-care serologic-based diagnostic tests, such as the Widal test, are also limited by low sensitivity and specificity (*5*). In addition to variable sensitivity and specificity, available testing for enteric fever might be cost prohibitive, resulting in underreporting of the true incidence of disease. Furthermore, because of public health allocations of resources focused on other infectious diseases, accurate surveillance remains an epidemiologic challenge. It is important to capture the effect of those infections to better prioritize preventive measures, including vaccine implementation (*3*,*4*).

Although geostatistical models estimate a high prevalence of enteric fever in sub-Saharan Africa, relatively few studies have provided direct disease burden estimates in the region (*2*,*8*). The available data suggest a very high burden of typhoid in Kenya; however, most studies focused primarily on the densely populated capital city of Nairobi and its surrounding areas, with limited data available from the western or coastal regions (*2*,*9*). Although the Kenya Ministry of Health offers typhoid vaccination for high-risk groups, it has not implemented a routine immunization series for all persons (*9*). To further understand the burden of typhoid in western and coastal region of Kenya, we implemented a serosurveillance tool to estimate the population-level enteric fever seroincidence rate and identify risk factors for infection among children.

## Methods

### Study Cohort

We used archived serum samples and accompanying survey data that evaluated the burden of chikungunya virus and dengue virus infections among children 2–18 years of age across 4 sites in Kenya (*10*). Although the parent study included a longitudinal cohort, this analysis is cross-sectional serosurvey using data and serum samples collected from a periodic sampling timepoint (April 2017–January 2018) and comprises a random subset of 1,408 children. Two geographically distinct areas in Kenya are represented in this analysis: coastal Kenya (Msambweni and Ukunda) and western Kenya (Chulaimbo and Kisumu). Those 2 areas have different baseline infrastructure (higher wealth in the west) and weather patterns (higher temperature and humidity and longer rainy seasons on the coast) (Appendix Table 1). We selected a rural town in each area, Msawbweni on the coast and Chulaimbo in the west, that had less infrastructure and fewer resources than its adjacent densely populated urban center (Appendix Table 2) (*10*). We recruited households by random enrollment within confined structured zones in each study community across a similar time period. We administered demographic surveys designed to collect information about the household, built infrastructure, and behavioral patterns related to mosquitoborne infections but also captured information relevant to food and waterborne illnesses, such as population density and access to piped water and latrines (*10*). The ethics review boards with the Kenya Medical Research Institute (approval no. SSC95 2611), Stanford University (approval no. 31488), and Mass General Brigham (approval no. 2019P000152) approved the study protocol.

### Sample Collection and Testing

We collected blood samples during the same household visit at which we administered surveys. We centrifuged blood samples and stored serum aliquots at −70°C until testing. We used 1 serum sample from each participant to measure hemolysin E HlyE IgG levels at Massachusetts General Hospital (Boston, MA, USA) by kinetic ELISA, as previously described (*6*).

### Statistical Analysis

We estimated seroconversion rates from cross-sectional serosurveys using models of HlyE IgG decay derived from blood culture–confirmed enteric fever cases (*6*). Those models account for peak antibody responses, decay rates, and variability in immune responses while incorporating multiple biomarkers, measurement noise, and cross-reactivity (*11*–*13*). We implemented our approach using the open-source R package serocalculator (https://cran.r-project.org/web/packages/serocalculator). We paired demographic information with the serologic results and analyzed for associations with age, population density, water source, latrine availability, and wealth. We calculated the population density using zonal statistics in QGIS version 3.28.9 (https://qgis.org), and obtained population counts from WorldPop (https://www.worldpop.org). We divided the population into quartiles representing increasing population density across the cohort. We calculated a wealth index by multiple correspondence analysis, using variables related to tangible assets (e.g., radio, motor vehicle, television, bicycle, telephone), house ownership and characteristics (e.g., number of rooms used for sleeping, persons per room, window screens, and building materials), and access to utilities and infrastructure (e.g., source of water, sanitation facility, and location) (*14**,**15*). We divided the scores into quartiles representing increasing socioeconomic status (SES) as a measure of wealth throughout the group (*14*). We performed all analysis in R version 4.4.3 (The R Project for Statistical Computing, https://www.r-project.org).

## Results

### Study Population

Of the 1,408 participants included in the study, 323 were from Kisumu (west), 323 from Chulaimbo (west), 299 from Ukunda (coast), and 473 from Msambweni (coast) (Table). The median age of participants was 10.6 (interquartile range [IQR] 7.8–13.1) years; the median age for each specific community was 10.2–10.9 years of age (Table). Wealth distribution and population density were higher in Kisumu and Ukunda than in Chulaimbo and Msambweni (Appendix Tables 1, 2).

**Table:** Characteristics of study sites and participants in study of typhoidal *Salmonella* in children, Kenya, 2017–2018*

Characteristic	Study site				Overall, n = 1,408
	Kisumu, n = 323	Chulaimbo, n = 323	Ukunda, n = 299	Msambweni, n = 473	
Sex					
F	173 (54)	146 (45)	166 (56)	225 (48)	719 (51)
M	150 (46)	177 (55)	133 (44)	248 (52)	689 (49)
Age, y					
2–5	32 (10)	27 (8)	20 (7)	47 (10)	126 (9)
6–10	140 (44)	146 (46)	138 (46)	227 (48)	651 (46)
11–18	147 (46)	145 (46)	141 (47)	199 (42)	632 (45)
Median (IQR)	10.2 (7.8–12.6)	10.7 (7.8–13.5)	10.5 (7.93–13.1)	10.9 (7.9–13.6)	10.6 (7.8–13.1)
Piped water, n = 1,382	300/303 (>99)	80/300 (26)	275/299 (92)	71/480 (14)	656 (47)
Latrine access, n = 1,371	301/301 (100)	294/300 (98)	296/297 (>99)	193/473 (41)	1,084 (79)
Location	West	West	Coast	Coast	

*Values are no. (%) except as indicated. IQR, interquartile range.

### Serology Findings

**Figure 1:**
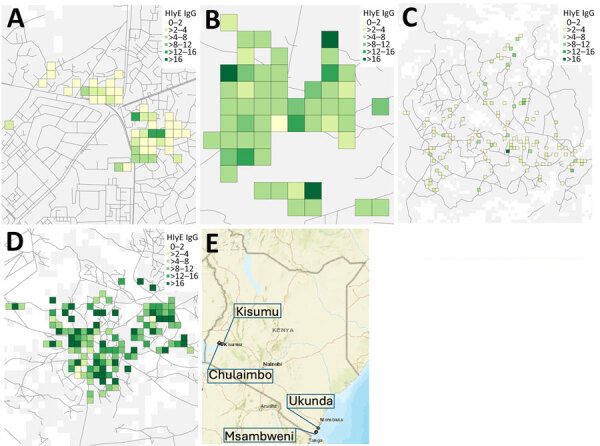
Sites used in study of seroincidence rate of typhoidal *Salmonella* in children, Kenya, 2017–2018. The serologic density of HlyE IgG, measured in ELISA units, was mapped onto study sites. Boxes represent a 100 m × 100 m grid. Kisumu (A) and Ukunda (B) are the more densely populated sites, Chulaimbo (C) and Msambweni (D) the less densely populated sites. E) Locations of study sites within Kenya.

**Figure 2:**
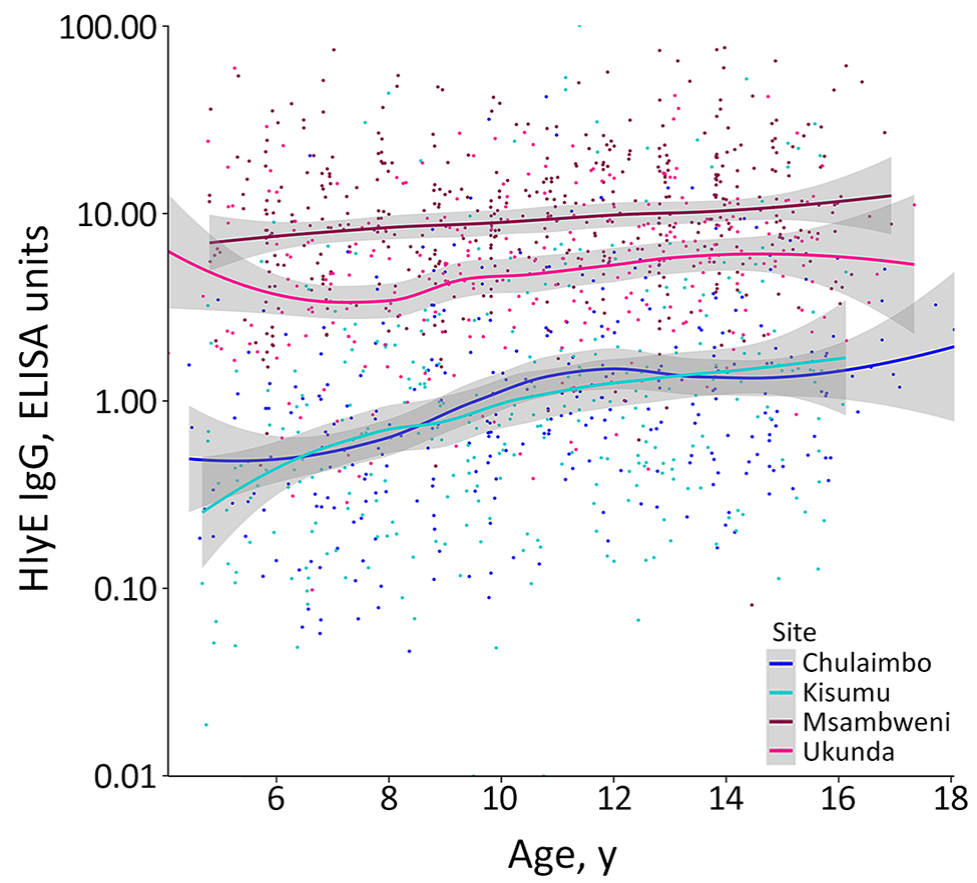
Antibody response by participant age and location in study of seroincidence rate of typhoidal *Salmonella* in children, Kenya, 2017–2018. Dots represent individual antibody responses, curves represent smoothed cumulative responses, and gray shading indicates 95% CIs.

We found higher HlyE IgG levels in the coastal population than the western population (Figures 1, 2). Median HlyE IgG level in the coastal population was 7.73 (IQR 4.10–10.97) ELISA units. Median HlyE IgG level in the western population was 0.86 (IQR 0.38–2.73) ELISA units. 

**Figure 3:**
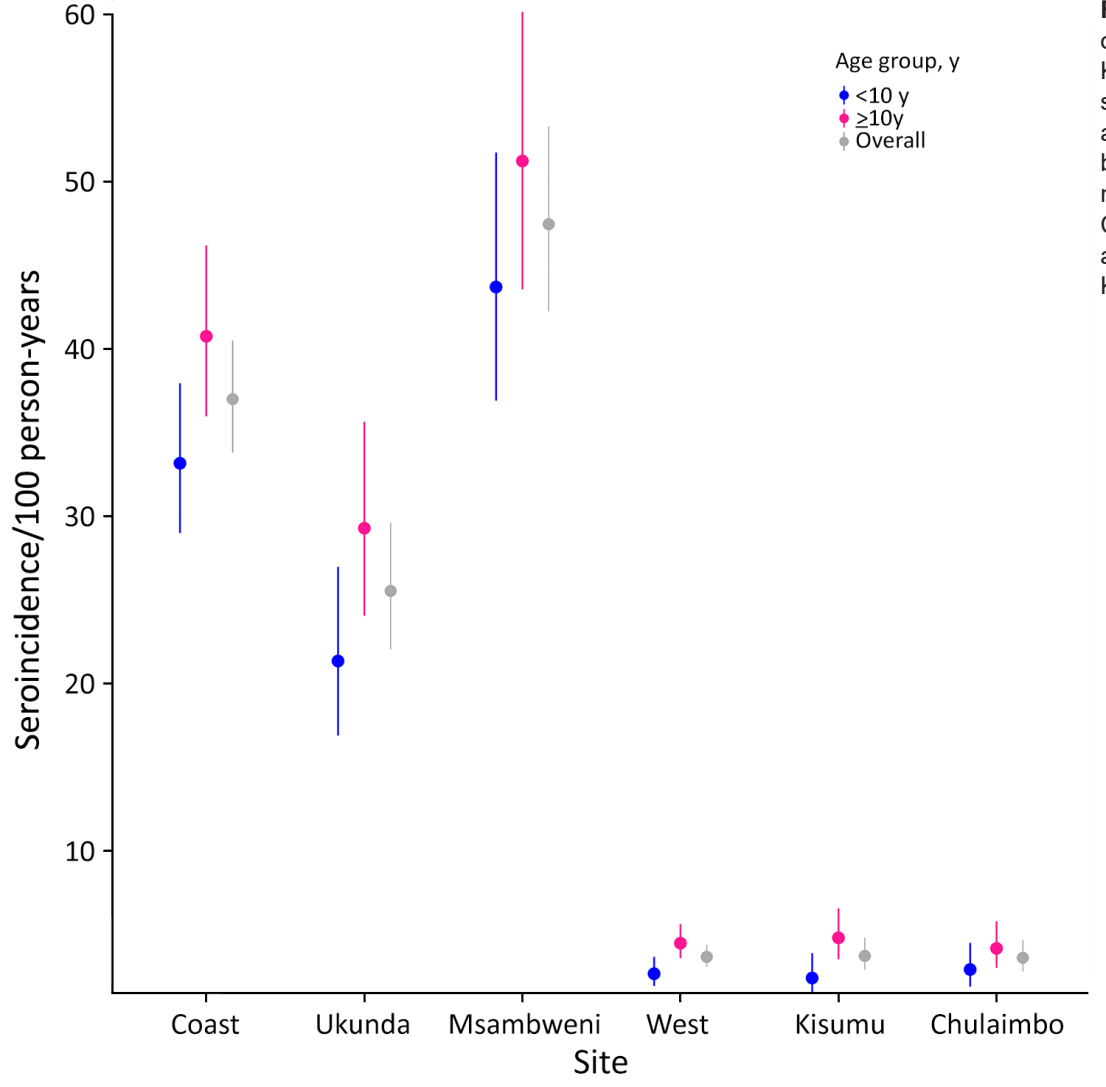
Seroincidence in study of typhoidal *Salmonella* in children, Kenya, 2017–2018. Typhoid seroincidence rate by region and study site is shown stratified by patient age. Dots represent medians; error bars indicate 95% CIs. Coast sites were Ukunda and Msambweni; west sites were Kisumu and Chulaimbo.

**Figure 4:**
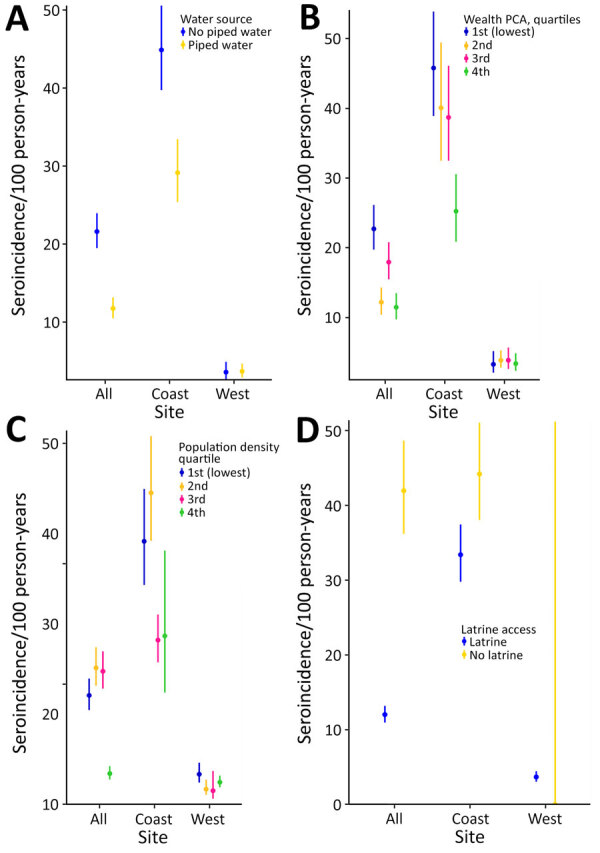
Typhoidal *Salmonella* seroincidence by site characteristics in study of typhoidal *Salmonella* in children, Kenya, 2017–2018. A) Seroincidence by water source. B) Seroincidence stratified by wealth. C) Seroincidence stratified by population density. D) Seroincidence by latrine type. Dots represent medians; error bars indicate 95% CIs. PCA, principal component analysis.

The overall seroincidence rate for the Kenya cohort was 9.1 (95% CI 8.4–9.8) per 100 person-years. In the coastal region, the seroincidence rate was 37 (95% CI 33.8–40.5) per 100 person-years, and in the western region, it was 3.6 per 100 person-years (95% CI 3.0–4.4). We found no significant difference when comparing seroincidence rates by age (<10 years or >10 years of age); however, there was a trend of higher seroincidence rates in the >10 years group (Figure 3). Piped water, higher wealth, latrine use, and urban location were associated with lower enteric fever seroincidence rates in the coastal region (Figure 4). No risk factors were significantly associated with seroincidence rates in the western region of Kenya.

## Discussion

In this study, we leveraged archived serum samples from a large arbovirus cohort study in Kenya to obtain population-level enteric fever seroincidence rates based on HlyE IgG responses. We found an estimated 10-fold higher seroincidence rate of typhoidal *Salmonella* in the coastal region than the western region. The enteric fever seroincidence rate we estimated in coastal Kenya is similar to the rate estimated in Bangladesh, where ongoing blood culture surveillance for enteric fever has confirmed a high prevalence of *Salmonella* Typhi and Paratyphi A (*16*). The seroincidence rate we estimated in the western region of Kenya is closer to that of Kathmandu, Nepal (5.8/100 person-years), where the typhoid conjugate vaccine campaign was launched in 2022 (*6*,*16*).

The enteric fever seroincidence rates we estimated (9.1/100 person-years or 9,100/100,000 person-years overall) exceed those from previous culture-based studies in the region, for which an incidence rate of >100/100,000 persons/year is considered high for typhoid fever. Previous clinical surveillance studies have estimated a range of incidence with an extrapolated crude incidence of 39/100,000 persons/year in eastern Africa (*17*); an adjusted incidence of 284/100,000 person-years of observation in Kibera, Kenya (*3*); and an estimated incidence of 620/100,000 person-years in eastern sub-Saharan Africa (*8*). We found no direct comparison to clinical blood culture incidence rates available from Kenya or East Africa (*18*). The seroincidence rate we observed in this study is comparable to rates reported in other regions where typhoidal *Salmonella* is recognized as a public health concern; vaccination campaigns are targeting those populations. As for many infectious diseases in sub-Saharan Africa, limited surveillance data can reduce allocation of resources to address those problems. We demonstrated a substantially higher seroincidence of typhoidal *Salmonella* in western and coastal regions of Kenya than other areas in the country that might have an unrecognized higher level of exposure. Causes of higher seroincidence could be the limited availability and affordability of diagnostics, low sensitivity of culture, and subclinical infections. 

Most typhoidal *Salmonella* studies evaluate febrile participants. Because patients can be exposed to *Salmonella* Typhi and Paratyphi without experiencing a symptomatic infection, the seroincidence rate in those studies will seem higher than in clinical studies because they included patients with subclinical infection otherwise not captured by clinical surveillance (*19*). Variable sensitivity in blood culture sampling and inoculum, especially in young children, could also cause false negative results. Furthermore, differences in health-seeking behavior can also account for delayed symptoms or missed typhoid cases in various communities (*20*–*22*).

In addition to estimating the population-level enteric fever seroincidence rate, we explored potential influence of established risk factors, including population density, SES, and water, sanitation, and hygiene measures (*23*,*24*). Consistent with previous studies, we found notable differences in seroincidence rates with water access, latrine use, and overall wealth. In the coastal region, we observed a trend toward a higher seroincidence rate of enteric fever associated with lower wealth, lower population density, and use of nonpiped water. In our study, we found higher enteric fever seroincidence rates in the coastal villages than in western villages. When evaluating the source of water, most nonpiped water was located in the coastal sites, which likely contributed to the higher levels of typhoidal *Salmonella* found in the coastal sites than the western sites. The coastal sites also have higher humidity and relative temperature and longer rainy seasons, which can be associated with foodborne and waterborne infections (*25*).

We also noted differences in seroincidence rates within the coastal sites and in comparison to the western sites. The less densely populated, less developed, and effectively rural site of Msambweni on the coast had the highest seroincidence rate, which deviates from studies in southern Asia and other parts of the world where denser populations have been associated with increased risk for infection. Many of those densely populated communities often do not have access to piped water, and residents live in housing with inadequate sanitation, which is different from our study sites (*26*). Msambweni had most of the participants of lowest SES from all 4 study sites and likely has multiple factors contributing to increased seroincidence rate, including lack of piped water, poor sanitation, and other environmental factors. Seasonal outbreaks or community sanitation leakages were possible but not reported during the study period. In contrast, we noted no major differences in the west between the urban center in Kisumu and its rural adjacent site, Chulaimbo. We attributed that finding to lower statistical power to detect a difference, given the low seroincidence rate overall in western sites; another possible cause is the difference in wealth index, population density, and environmental factors between the geographic sites. We noted a greater divide in calculated SES quartiles in the coastal region than in the west, where the distribution was closer, as was the calculated seroincidence rate. Last, the differing climate and susceptibility to flooding on the coast can also contribute to the higher seroincidence rate found in the coastal region in this study (*27**,**28*).

Our findings demonstrate that the risk for exposure and burden of typhoidal *Salmonella* is not homogenous and varies greatly both between regions and within populations in the same country. Previous studies have been performed in dense urban slums, which have specific factors that contribute to propagation of infection (*3**,**29*). When comparing urban and rural settings, the wide variation in living conditions, wealth, and access can influence exposure to typhoidal *Salmonella* pathogens. Our surveys did not capture the possibility of sanitation leakages, which can contribute to typhoid exposure. Of note, no major outbreaks were reported during the study period.

The 2 main risk factors we explored in this study, water source and latrine access, were also included in the wealth index calculation and trended in the same direction as wealth, likely influencing the trend we identified. In addition, the original study (*10*) focused on mosquitoborne infections and did not include a comprehensive assessment of all the risk factors associated with enteric fever infection. For testing, HlyE is expressed by both *Salmonella* Typhi and *Salmonella* Paratyphi A; therefore, antibody responses to the antigen cannot distinguish between infections caused by these 2 pathogens (*2*). Although *Salmonella* Paratyphi A is a common cause of enteric fever in Asia, it is considered rare in Africa (*30*,*31*). Although HlyE is also present in the genomes of *Shigella* species and *Escherichia coli,* its expression during infection with those pathogens likely differs and may be repressed or disrupted in some lineages (*32*). The sample size might not be sufficient to comprehensively represent the greater regions across Kenya. In addition, although a sample size of 300–400 may be sufficient for calculating the seroincidence rate, those estimates are limited when stratifying by age and other typhoid-associated risk factors and should be explored further in a larger study. Furthermore, with random nonstratified sampling, we observed fewer children in the <5 years age group, which also can influence the overall seroincidence rate. Last, the samples were collected in 2017–2018; the seroincidence rate might have changed since that time given different seasons, climate change, drought/flooding, and other factors like the COVID-19 pandemic, which caused changes in movement and behavior. More detailed incidence studies are needed to improve incidence estimates to reveal the comprehensive burden of infection for implementation of public health measures and to determine if the burden remains high in Kenya.

Despite those limitations, our study demonstrates that the enteric fever seroincidence rate is high in Kenya, particularly in the coastal region, where incidence rates were comparable to other highly endemic areas for typhoid in Asia (e.g., Dhaka, Bangladesh) and >100-fold higher than estimates by blood culture surveillance. Our findings suggest there is a role for implementing typhoid conjugate vaccine to additional populations in coastal and western Kenya, in addition to the current practice of provide the vaccine to high-risk groups.

Appendix Additional information about typhoidal *Salmonella* seroincidence in children, Kenya, 2017–2018.
