# Supplementary material for: Seroincidence Rate of Typhoidal Salmonella in Children, Kenya, 2017–2018
Source: Emerg Infect Dis. 2026 Mar;32(3):368–75. doi: 10.3201/eid3203.250469 (PMC13016032; doi:10.3201/eid3203.250469)
Supplement: Appendix — Additional information about typhoidal Salmonella seroincidence in children, Kenya, 2017–2018. [file 25-0469-Techapp-s1.pdf]

Article DOI: https://doi.org/10.3201/eid3203.250469

EID cannot ensure accessibility for supplementary materials supplied by authors. Readers who have difficulty accessing supplementary content should contact the authors for assistance.

Seroincidence Rate of Typhoidal *Salmonella* in Children, Kenya, 2017–2018

Appendix

Appendix Table 1. Wealth index with percentages by quartile in study of seroincidence rate of typhoidal *Salmonella* in children, Kenya, 2017–2018*

Quartile	Kisumu, n = 301	Chulaimbo, n = 308	Ukunda, n = 297	Msambweni, n = 473	Overall, n = 1,371
Wealth index, top quartile (4)	18% (63)	4% (13)	55% (186)	22% (76)	24.7% (338)
Wealth index, middle quartile (3)	47% (162)	18% (61)	18% (62)	13% (62)	25.3% (347)
Wealth index, middle quartile (2)	22% (75)	49% (169)	10% (36)	18% (63)	25% (343)
Wealth index, lowest quartile (1)	<1% (1)	17% (57)	4% (13)	79% (272)	25% (343)

*Factors included in the wealth calculation were house size, house crowding, window screens, bednet ownership, type of floor material, roof type, cooking fuel, water source, light source, land ownership, latrine availability, latrine location, presence of a domestic worker, and ownership of a TV, telephone, radio, bicycle, and motor vehicle.

Appendix Table 2. Population density per 100 m radius with percentages by respective quartiles, in study of seroincidence rate of typhoidal *Salmonella* in children, Kenya, 2017–2018.

Quartile	Kisumu, n = 303	Chulaimbo, n = 308	Ukunda, n = 284	Msambweni, n = 470	Overall, n = 1,365
Population density, top quartile (4)	90% (301)	(0)	10% (35)	(0)	24.6% (336)
Population density, middle quartile (3)	<1% (2)	12% (40)	74% (249)	14% (47)	24.8% (338)
Population density, middle quartile (2)	(0)	33% (112)	(0)	67% (232)	25.2% (344)
Population density, lowest quartile (1)	(0)	45% (156)	(0)	55% (191)	25.4% (347)
